# Evaluation of Breath-Holding Test in Assessment of Peripheral Chemoreflex Sensitivity in Patients with Chronic Heart Failure

**DOI:** 10.2174/1874306401711010067

**Published:** 2017-12-27

**Authors:** Nikita Trembach, Igor Zabolotskikh

**Affiliations:** Kuban State Medical University, Department of Anesthesiology, Reanimatology and Transfusiology. Krasnodar, Russian Federation

**Keywords:** Peripheral chemosensitivity, Voluntary breath-holding, Single-breath carbon dioxide test, Reliability, Chemoreflex, Chromic Heart Failure

## Abstract

**Background:**

The sensitivity of peripheral chemoreflex is a marker of the severity of heart failure and the prognosis of the outcome in these patients. The assessment of chemosensitivity in these patients remains an actual problem.

**Objective:**

The aim of the study was to explore the relationship between a Breath-Holding Test (BHT) and single-breath carbon dioxide test and to evaluate the reliability of both tests in patients with Heart Failure (HF).

**Method:**

The study was performed in 43 patients with chronic heart failure. All subjects underwent BHT and single-breath carbon dioxide (CB-CO_2_), the evaluation of both tests was repeated a month later. Relationship of two test was evaluated by correlation analysis. Reliability was assessed with calculation of Standard Error of Measurement (SEM), Coefficient of Variation (CV) and Intraclass Correlation Coefficient (ICC).

**Results:**

The duration of the breath-holding was inversely correlated to the result of CB-CO_2_ test (r = -0.86 at first measurement and r = -0.79 after a month) The ICC was 0.87 (95%CI: 0.78–0.93) for SB-CO_2_ test and 0,93 (95%CI: 0.88–0.96) for BHT, the CV was 24% for SB-CO_2_ and 13% for BHT. SEM for SB-CO_2_ test was 0.04 L / min / mmHg and limits of variation was 0.11 L / min / mmHg; SEM for BHT was 3.6 sec and limits of variation was10 sec.

**Conclusion:**

Breath-holding test is a reliable and safe method for assessing the sensitivity of peripheral chemoreflex to carbon dioxide in patients with heart failure.

## 1 INTRODUCTION

The problem of impaired sensitivity of peripheral chemoreflex in patients with chronic cardiorespiratory diseases has given increased attention in the recent decades. The state of chemosensitivity is not only a marker of the severity of violations of reflex regulation in heart failure, but also a good prognostic marker, reflecting the likelihood of an adverse outcome [1, 2]. The pathogenesis chemoreflex hypersensitivity in Heart Failure (HF) is rather complicated and has not been fully studied. Earlier studies indicated that activation of the local angiotensin II system [3, 4] and a decreased neural nitric oxide synthase–nitric oxide pathway [5, 6] in the carotid bodies are associated with chemoreceptor hyperactivity during progression of HF. It was also observed, that decreased blood flow in carotid bodies contributes to chemoreflex dysfunction [7]. An increase in the afferent flow from the peripheral chemoreceptors leads to an increase in sympathetic tone due to stimulation of the autonomic centers in the medulla oblongata [8] and hypothalamus [9]. These disorders lead to an increased risk of cardiac adverse events and a poor outcome in patients with HF.

One of the most useful methods in clinical practice for assessing the sensitivity of peripheral chemoreflex is the Single-Breath Carbon Dioxide (SB-CO_2_) test, which evaluates the response of the respiratory system to a single inhalation of a gas mixture with a high content of carbon dioxide [10-12]. Test showed good accuracy in subjects without chronic diseases [11, 12] and in patients with heart failure [13]. Unfortunately, although this method is simpler and safer than hypoxic tests, it has several disadvantages. Possible variability in patients with HF and the need for complex equipment can limit the routine application of this method. Moreover, this method has a poor absolute reliability [13].

Recent studies have shown that the duration of a voluntary breath-holding during a Breath-Holding Test (BHT) indirectly reflects the sensitivity of peripheral chemoreflex to carbon dioxide in subjects without cardiac and respiratory diseases [14]. However, its reproducibility and relationship with SB-CO_2_ test was not evaluated in patients with HF previously.

The aim of the study was to explore the relationship between a breath-holding test and single-breath carbon dioxide test and to evaluate the reliability of both tests in patients with chronic heart failure.

## 2 METHODS

### 2.1 Study Population

The study was conducted in patients with chronic heart failure of 2-3 functional class who were treated in the department of cardiology. Patients with respiratory diseases, central nervous system diseases and mental disorders were not included in the study. The permission of the Ethics Committee of Kuban State Medical University was obtained for the work in February 2017, all participants signed an informed consent for the tests.

### 2.2 Measurements

Spirometry and blood gas analysis, Brain Natriuretic Peptide (BNP) assessment, blood pressure and heart rate (from electrocardiography) assessment and echocardiography were performed in all patients after the admission to the department the day before SB-CO_2_ test.

In all participants, BHT and SB-CO_2_ test were performed in the morning before fasting.

The single-breath carbon dioxide test was performed as follows [14]. After quiet breathing by atmospheric air, one inhalation was carried out with a hypercapnic mixture (imperceptible to the subject for objectification of the test), after which the valve was placed on the regime with respiration by atmospheric air. This test was repeated 10 times with a break for two minutes. The sensitivity of peripheral chemoreflex was defined as the ratio of the difference in the minute ventilation to the difference in the partial pressure of end-tidal carbon dioxide (L / min / mmHg). The average value for 10 tests was calculated and this average was recorded as the individual sensitivity of peripheral chemoreflex to carbon dioxide.

The breath-holding test [15] was carried out on a next day at the same time. After an inhalation of atmospheric air volume, which was equal to approximately two-thirds of the subject's vital capacity, a voluntary maximal inspiratory breath-holding was performed. The counting of the duration of the breath-holding was made by a stopwatch from the beginning of the inspiration to the appearance of reflex contractions of the diaphragm, which were determined by the palm of the researcher located in the epigastric region of the subject. The test was performed three times with an interval of 10 minutes. The mean value of the three samples was recorded as a result of BHT.

A month later, both tests were performed repeatedly in all subjects under similar conditions and on the same principles by same researcher.

### 2.3 Statistical Analysis

We tested the hypothesis of normality for all variables with Shapiro-Wilk test. Due to the normal distribution, the data are presented as mean ± standard deviation. To evaluate the relationship between the results of the two tests, we calculated the Pearson’s correlation coefficient (r).

To assess absolute reliability, we used the SEM (Standard Measurement Error) calculation, which reflects the intra-subject variability [16]. Using this parameter, we determined the 95% limits of random variation between the two measurements (±2.77×SEM). In order to compare the results of our study with the results of other studies, we also calculated the coefficient of variation (CV) [17]. To estimate the relative reliability, we calculated the Intra-class Correlation Coefficient (ICC) with 95% confidence intervals [16].

## 3 RESULTS

Demographic and clinical characteristics of the study population are reported in Table (**1**). 43 patients were included in study. The data presented in the table indicate that in patients the spirometry and blood gas composition were within the normal range.

**Fig. (1):**
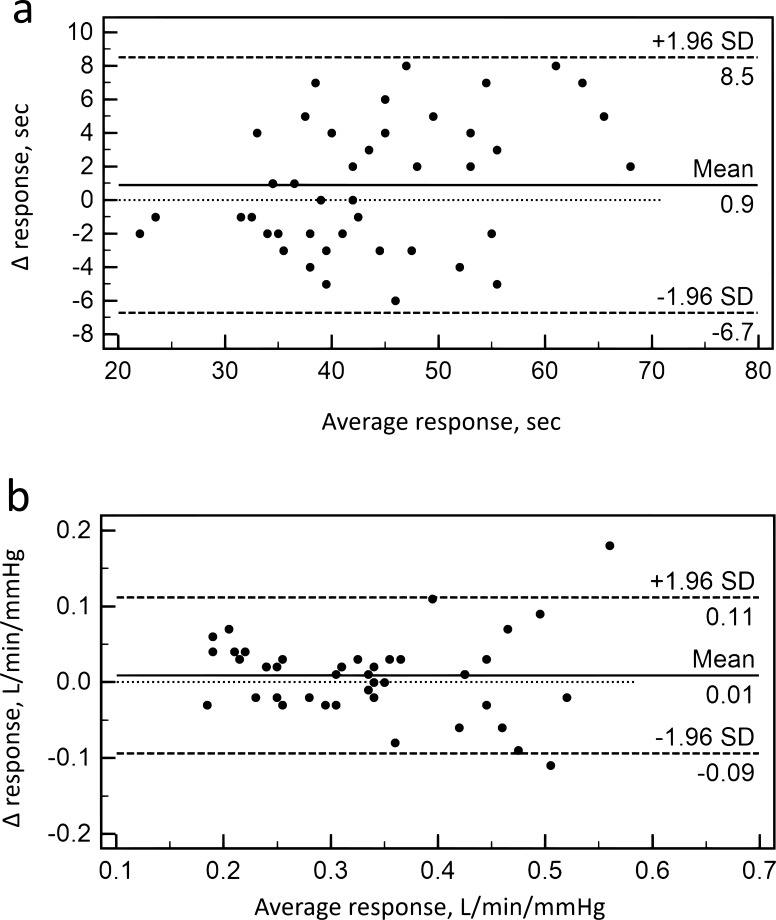
Bland–Altman plot of the difference between the results of two BHT (a) and two SB-CO_2_ test (b) against their average.

When analyzing the Bland-Altman diagrams, more than 95% of the data points were within ± 2SD of the mean difference for BHD and SB-CO_2_ test (Fig. **1**). There were no systemic changes between two measurements in both tests.

**Fig. (2):**
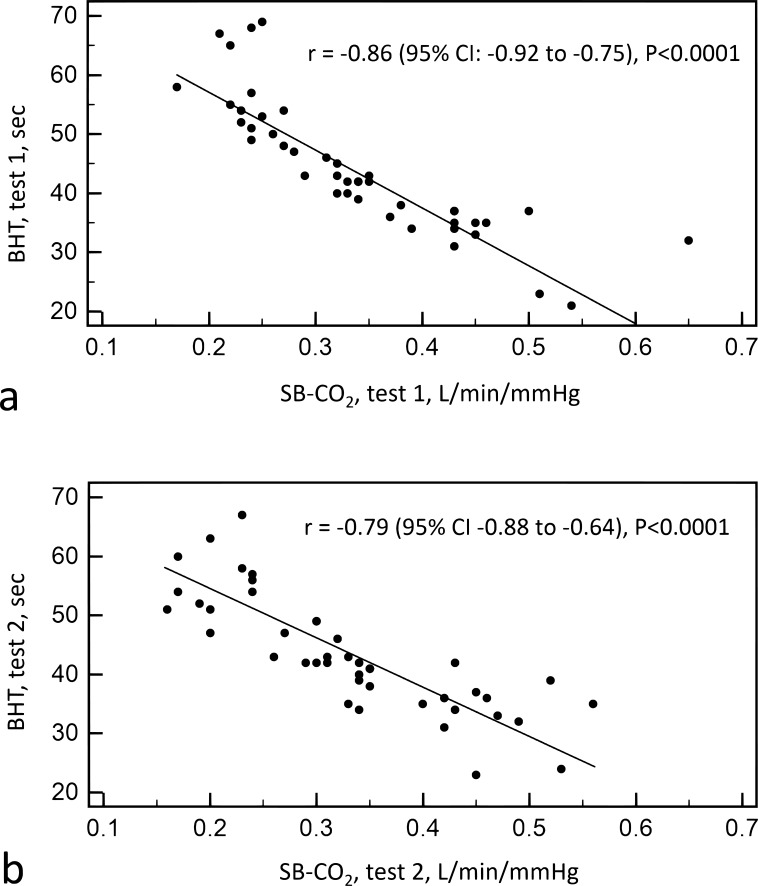
Correlation between two tests at first measurement (a) and after month (b) SB-CO2 -single-breath carbon dioxide test, BHT- breath-holding test.

A strong inverse correlation between duration of breath-holding duration result of CB-CO2 test was observed (r = -0.86 (p<0.05) at first measurement and r = -0.79 (p<0.05) after a month) (Fig. **2**).

Mean coefficients of variation for SB-CO_2_ test was 24% and ranged between 0% and 36%. Mean coefficients of variation for BHT was 13% and ranged between 0% and 21%. The ICC was 0.87 (95%CI: 0.78–0.93) for SB-CO_2_ test and 0,93 (95%CI: 0.88–0.96) for BHT, indicating substantial relative reliability for both tests. The parameters estimating the reliability of SB-CO_2_ and BHT are presented in (Table **2**).

## 4 DISCUSSION

One of the main results of our study is the fact that the duration of a voluntary breath-hold duration in patients with CH depends on the sensitivity of peripheral chemoreceptors to carbon dioxide. The data obtained by us showed that the duration of a voluntary threshold apnea has a strong inverse correlation with the ventilatory response to the SB-CO_2_ test, both at the first measurement and after a month. These data are consistent with the results of our previous studies in which we found this pattern in healthy subjects [14]. The mean duration of breath-holding test was 44 and 43 seconds, which is somewhat less than the values obtained earlier in healthy subjects with a similar technique. The mean sensitivity of peripheral chemoreceptors to carbon dioxide was higher than the values obtained earlier [13] in a study in patients with chronic heart failure. Differences in the results appear to be differences in the study groups, since the presence of CH does not necessarily mean the presence of a violation of autonomic regulation, and these disorders are a marker of the progression of the disease and the risk of an adverse outcome [18].

In this study, we determined the relative and absolute reliability of the two tests.

The data obtained show that SEM for SB-CO2 test was 0.04 L / min / mmHg and LoV was 0.11 L / min / mmHg, which is approximately 33% of the mean value of all the results obtained for this test. Clinically, it means that the minimum difference to be confident of real patient change would be 0.11 L / min / mmHg. Relative reliability of this method in our study was higher than that obtained in earlier studies on the sensitivity of peripheral chemoreflex in patients with CH [13], in which the absolute value of LoV was comparable (0.15 L / min / mmHg) to that obtained by us, but the mean value of the ventilatory response was lower. SEM for BHT was 3.6 seconds, and LoV - 10 seconds. This means that a second measurement would need to differ from the first by more than 10 sec for a therapist to be sure that the difference was in the 2 measurements. LoV, determined by us is less than 30% of the mean value, which confirms the good absolute reliability of the method.

The ICC for SB-CO_2_ test was 0.87, which is regarded as very good reliability, and ICC for the breath-holding test was even higher (0.93), which is regarded as perfect reliability [19]. This means that both tests are suitable for assessing the ratio of the subject to a particular group, that is, determining whether there is or not a disturbance in the sensitivity of the peripheral chemoreflex [20].

When comparing CV for SB-CO_2_, we obtained a reproducibility of 24%, which is consistent with the data obtained in the study in patients with CH (20.4%) [13] and in healthy people (17%) [12]. Due to available literature, we made a first attempt to assess the reliability of BHT in patients with CH by evaluating SEM and ICC. The CV we calculated was 13%, consistent with the data obtained during the study of the reproducibility of the method in healthy subjects [14]. Strong inverse correlation between the two methods and good reliability suggest that the relationship between the sensitivity of peripheral chemoreflex and the duration of a voluntary apnea in patients with CH is not compromised.

The duration of the voluntary breath-holding depends on several factors that were not taken into account in our study. Although peripheral chemoreceptors are included in the first line, and central chemoreception is a minor determinant of breath-holding duration [21], it is impossible to exclude the contribution of central chemoreceptors, especially with prolonged apnea. Also the duration of breath-holding depends on the metabolic rate, the initial blood gases [22], which was not taken into account in our study, although the influence of these factors, we believe, was minimized. It should be noted that the influence of these factors is also significant when using more valid methods. Undoubtedly, a large contribution to the duration is made by the initial volume of the lungs at the peak of inspiration [23], the objectification of this volume makes it more accurate and reproducible. The presence of respiratory diseases can significantly change the biomechanics of respiration and contribute to the result of the BHT, so the presence of these diseases was the criterion of exclusion. The question of the possibility of evaluating peripheral chemoreflex in conditions of respiratory disorders requires a separate study.

So, BHT is not a method of quantitative evaluation of peripheral chemoreflex sensitivity, however, the presence of a strong relationship between the duration of breath-holding and the sensitivity of chemoreceptors allows a quick assessment of the function of the cardiorespiratory reflexes. BHT also can be helpful in evaluation of the of therapy effectiveness, since the sensitivity of peripheral chemoreflex is a promising treatment target.

## CONCLUSION

Breath-holding test is a reliable and safe method for assessing the sensitivity of peripheral chemoreflex to carbon dioxide in patients with chronic heart failure.

## Figures and Tables

**Table 1:** Demographic and clinical characteristics of the study population (n=43).

Parameter	Value
Age, years	49±4
Weight, kg	73.6±4.0
Height, cm	177±6
BMI, kg/m^2^	23.5±1.5
NYHA class	2.3±0.4
LVEF, %	34±8
BNP, pg/ml	112±34
Heart rate, min -1	87±12
SBP, mmHg	145±23
DBP, mmHg	87±14
SaO_2_,%	98.6±1.1
PaCO_2_, mmHg	35.8±1.5
PaO2, mmHg	86.8±4.7
VC, % predicted	97.7±3.3
FVC, % predicted	95.0±2.6
FEV_1_, % predicted	95.0±2.3
FEV_1_/FVC, % predicted	0.98±0.05
Breathing rate, min^-1^	11.3±2.1
Medical therapy	
ACE-I/ARB, %	98
ß-blockers, %	100
Aldosterone antagonists, %	88
Loop diuretics, %	35
Thiazide diuretics, %	40
Statins, %	79

BMI – body mass index, NYHA – New-York Heart Association, LVEF – left ventricle ejection fraction, BNP – brain natriuretic peptide, SBP – systolic blood pressure, DBP - diastolic blood pressure, SaO_2_ – oxygen saturation, PaCO_2_ - arterial carbon dioxide partial pressure, PaO_2_ - arterial oxygen partial pressure VC - vital capacity, FVC – forced vital capacity, FEV_1_ – forced expiratory volume in a first second, ACE-I/ARB - angiotensin-converting enzyme inhibitor/angiotensin receptor blocker

**Table 2:** Descriptive and agreement statistics for both tests

	Test 1	Test 2	Difference	SEM	95%LoV	CV,%	ICC (95% CI)
SB-CO_2_, L/min/mmHg	0.34±0.13	0.33±0.15	0.01± 0.008	0.04	-0.11-0.11	24	0.87 (0.78 - 0.93)
BHT, sec	44±11	43±14	0.9±0.6	3.6	-10-10	13	0.93 (0.88 - 0.96)

SB-CO_2_ -single-breath carbon dioxide test, BHT- breath-holding test, LoV –limits of random variation, SEM- standard error of measurement, CV – coefficient of variation, ICC - intraclass correlation coefficient.

## LIST OF ABBREVIATIONS

BHT: = Breath-holding test
BMI: = Body mass index
BNP: = Brain natriuretic peptide
CV: = Coefficient of variation
DBP: = Diastolic blood pressure
FEV_1_: = Forced expiratory volume in a first second
FVC: = Forced vital capacity
HF: = Heart failure
ICC: = Intraclass correlation coefficient
LoV: = Limits of random variation
LVEF: = Left ventricle ejection fraction
NYHA: = New-York Heart Association
PaCO_2_: = Arterial carbon dioxide partial pressure
PaO_2_: = Arterial oxygen partial pressure
SB-CO_2_: = Single-breath carbon dioxide test
SBP: = Systolic blood pressure
SEM: = Standard error of measurement
SaO_2_: = Oxygen saturation
VC: = Vital capacity
